# Usefulness of Mendelian Randomization in Observational Epidemiology

**DOI:** 10.3390/ijerph7030711

**Published:** 2010-02-26

**Authors:** Murielle Bochud, Valentin Rousson

**Affiliations:** University Institute of Social and Preventive Medicine, Rue du Bugnon 17, 1005 Lausanne, Switzerland; E-Mail: Valentin.Rousson@chuv.ch

**Keywords:** genetic epidemiology, causality, observational studies, instrumental variables

## Abstract

Mendelian randomization refers to the random allocation of alleles at the time of gamete formation. In observational epidemiology, this refers to the use of genetic variants to estimate a causal effect between a modifiable risk factor and an outcome of interest. In this review, we recall the principles of a “Mendelian randomization” approach in observational epidemiology, which is based on the technique of instrumental variables; we provide simulations and an example based on real data to demonstrate its implications; we present the results of a systematic search on original articles having used this approach; and we discuss some limitations of this approach in view of what has been found so far.

## 1. Introduction

Observational studies have brought important insight into disease etiology. During the past decade however, the validity of observational studies has been questioned [1]. This is due to the fact that the role of selected risk, or protective, factors identified via observational studies could not be confirmed by subsequent large randomized controlled trials. For instance, hormonal replacement therapy appeared to protect women against coronary heart disease in observational studies [2], whereas randomized trials showed no such protection [3]. Other examples are given by antioxidant vitamin supplementation [4–6].

One cannot, for ethical and technical reasons, randomize risk factors using controlled trials in humans. The identification of risk factors therefore relies on observational studies, which are prone to spurious results due to confounding factors, reverse causation, and/or selection biases [7]. As a consequence, it is difficult to firmly establish causal relationships between risk factors and disease. Most common diseases (e.g., cancer, cardiovascular disease, *etc*.) are complex and are influenced by multiple risk factors that may be correlated with each other. In this context, each factor is expected to have a small influence on disease risk. Epidemiologists have the hard task to determine whether a putative risk factor is causally related to a specific disease, independently of all other risk factors. A promising approach to help epidemiologists in this task is Mendelian randomization. In this review, we first recall the principles of a “Mendelian randomization” approach in observational epidemiology (Section 2), we then provide some technical explanation of the method of instrumental variable (Section 3), followed by simulations and an example with real data (Section 4). We then present the results of a systematic search on original articles having used this approach (Section 5), discuss its limitations (Section 6) and present concluding remarks (Section 7).

## 2. Mendelian Randomization in Observation Epidemiology

Mendelian randomization refers to the random allocation of alleles at the time of gamete formation. A specific genotype carried by a person therefore results from two such randomized transmissions, one from the paternally inherited allele and the other from the maternally inherited allele. A logical consequence of these randomizations is that genotypes are not expected to be associated with known (measurable or not) or unknown confounders for any outcome of interest, except those lying on the causal pathway between the genotype and the outcome. This should hence allow analyzing the genotype-risk factor association and the genotype-outcome association in an unconfounded manner. By combining appropriately the results of these two analyses, one can get an estimate of the risk factor-outcome association, which is itself not confounded. This is analogous to randomized controlled trials (of sufficient sample size), in which the random allocation of treatment (or preventive measure) is expected to lead to an even distribution of (known or unknown) confounding factors across each groups. The term “Mendelian randomization” is now frequently used in observational epidemiology to refer to the use of genetic variants to estimate a causal effect between a specific modifiable risk factor and a trait/disease of interest. The idea is to overcome some of the problems encountered in observational epidemiology, such as residual confounding and reverse causation, by taking advantage of the natural random allocation of alleles during meiosis [8].

We here provide an example to illustrate this approach. The aldehyde dehydrogenase 2 (*ALDH2*) gene encodes the enzyme aldehyde dehydrogenase, which catalyzes the chemical transformation from acetaldehyde to acetic acid. Carriers of the ALDH2 *2*2 genotype have reduced alcohol consumption because of adverse reactions (facial flush, headache, nausea and drowsiness) due to acetaldehyde accumulation. This fact has been used to show that alcohol intake increases the risk of esophageal cancer [9] or head and neck cancer [10], which is consistent with the findings from observational studies. Whereas reported alcohol consumption may be subject to measurement errors, *ALDH2* genotypes can be measured accurately, are present since birth, result from the random allocation of the paternally and maternally inherited alleles, are strongly associated with alcohol consumption, and therefore provide a unique opportunity to assess, in an unconfounded manner, the risk of disease associated with alcohol consumption. As we shall discuss in Section 6, such an approach - although appealing - also raises some methodological issues.

**Figure 1.**
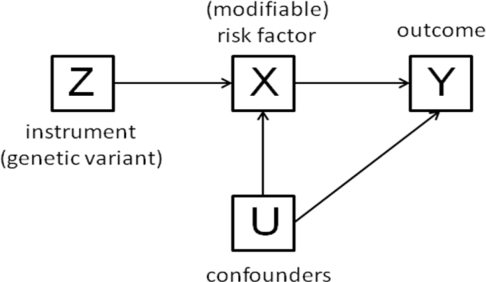
Directed acyclic graph (DAG) representing causal relationships between the genetic instrument (*Z*), the modifiable risk factor (*X*), the outcome (*Y*) and the (known or unknown, measurable or non-measurable) confounders (*U*), which satisfy the assumptions required by a Mendelian randomization. In a DAG, a node represents a variable and an arrow a direct causal effect. Because a cause must precede an effect, no cycle is allowed and this is why the graph is termed acyclic (there is no loop from one node back to itself following the arrows). See Greenland *et al.* [16] for more details on DAG.

Historically, the first description of the concept of Mendelian randomization in observational epidemiology is attributed to Katan [11], who suggested to use the *APOE* gene to infer causality between cholesterol and cancer. The concept was further developed by Davey Smith and Ebrahim [7,8,12,13], who have shown that the causal effect of a risk factor (X) on an outcome (Y) can be estimated by combining the effects of a genetic variant (Z) on X and on Y, provided that certain assumptions are met (see Figure 1). Thomas and Conti [14] have shown that the Mendelian randomization approach was in fact an application of the instrumental variable approach, which had been used since more than 70 years by econometricians. Wehby *et al.* have recently advocated that the term “Mendelian randomization” should be replaced by “instrumental variable analysis with genetic instruments” [15]. We tend to agree with this latter statement after having reviewed the medical literature and observed that the term “Mendelian randomization” was used with different meanings by different researchers, which might be confusing.

## 3. The Method of Instrumental Variables

We consider the case where an association between a continuous (or binary) modifiable exposure *X* and a continuous response *Y* is measured via a beta coefficient in a linear regression, defined as the average increase in *Y* when *X* is increased by one unit (respectively, when changing the category of *X* if the exposure is binary). When observing such an association in epidemiological research, however, it is often difficult to determine which of the two variables (*X* or *Y*) is the cause and which the effect, or whether a third variable (a confounder, *U*) related to both variables is responsible for the observed association. Moreover, measurement error could attenuate the beta coefficient. Thus, it is not obvious how a significant non-zero (e.g., positive) beta coefficient obtained from a classical (ordinary) least squares estimate should be interpreted. Here are five possible interpretations (among many others):

1. The beta coefficient is a consistent estimate of the causal effect of *X* on *Y*.
2. The beta coefficient is actually underestimating the true causal effect of *X* on *Y* because of measurement error.
3. The beta coefficient is overestimating the true causal effect of *X* on *Y* because of the presence of a confounder which is positively related to both *X* and *Y*.
4. The non-zero beta coefficient is entirely due to the presence of a confounder which is related to both *X* and *Y*: in fact there is no causal effect of *X* on *Y*.
5. The beta coefficient is non-zero because of a causal effect of *Y* on *X*, not of *X* on *Y* (*i.e*., reverse causation).

In other words, if the interest lies in assessing “the causal effect of *X* on *Y*”, *i.e.,* the effect that would be observed if one could intervene and change someone’s *X* level by one unit, leaving other characteristics unchanged, no definitive conclusion can be drawn from such an analysis. We shall see below, illustrated in the context described by Figure 1, how the method of instrumental variables can help in this regard.

A linear model (consistent with Figure 1) is given by:

$$Y=\alpha_{1}+\beta_{1}X+\gamma_{1}U$$

where *β*_1_ is the causal effect of *X* on *Y* and where *γ*_1_*U* plays the role of the error term, *U* being some unobserved confounder. Whenever *X* is correlated with the error term (see Figure 1), the expectation of the least squares estimate of the slope in this model, which we denote by $\beta_{1}^{LS}$, will be different from *β*_1_.

The method of instrumental variables has been proposed to correct for the bias of the least squares estimate. For this, we need to have at our disposal an “instrumental variable”, or instrument *Z*, for the time being continuous or binary, satisfying the following conditions: (1) *Z* is correlated with X, (2) *Z* is independent from *U*, and (3) *Z* and *Y* are independent given *X* and *U*. Note that the former of these conditions is verifiable from the data, whereas the latter two are largely not.

A second linear model (consistent with Figure 1) is then as follows:

$$X=\alpha_{2}+\beta_{2}Z+\gamma_{2}U$$

where *γ*_2_*U* plays the role of the error term in the model. Since *Z* is by assumption uncorrelated with this error term, the coefficients of this second model are estimated without bias by least squares. Note that the first model can be rewritten as:

$$Y=\alpha_{1}+\beta_{1}\alpha_{2}+\beta_{1}\beta_{2}Z+(\gamma_{1}+\beta_{1}\gamma_{2})\text{U}$$

Denoting *α*_3_ = *α*_1_ + *β*_1_*α*_2_, *β*_3_ *= β*_1_*β*_2_ and *γ*_3_ = *γ*_1_ + *β*_1_*γ*_2_, we obtain hence a third linear model:

$$Y=\alpha_{3}+\beta_{3}Z+\gamma_{3}U$$

where *γ*_3_*U* is the error term. Since *Z* is by assumption uncorrelated with this error term, the coefficients of this third model are also estimated without bias by least squares.

At the end, the parameters of the first model can be consistently estimated using relationships *α*_1_ = *α*_3_ − *β*_1_*α*_2_ and *β*_1_ = *β*_3_/*β*_2_, the denominator *β*_2_ being non zero by assumption. In particular, the instrumental variable (IV) estimate of the causal effect *β*_1_ in the first model is the quotient of the two least squares estimates of slope parameters *β*_3_ and *β*_2_ in the third and second models. Since the expectation of a quotient of two estimates is asymptotically equal to the quotient of the expectations of these estimates, the IV estimates are asymptotically unbiased, but they may be biased in finite samples.

Asymptotically, the IV estimates are normally distributed and explicit formulae for the standard errors are available, enabling to calculate confidence intervals and to test for the nullity of the causal effect *β*_1_ in the first model (as calculated e.g., with the ivregress 2sls command implemented in Stata 10.0). The standard error of the estimates will depend, among others, on the percentage of explained variance in the second model (itself related to the percentage of explained variance in the third model). If this percentage is low, the instrument is said to be weak, the standard errors will be large and the test above will have low power. Moreover, the bias of the IV estimates is typically larger, and the asymptotic normal distribution of the IV estimates may be a poor approximation to the true distribution, when the instrument is weak, the inference being then unreliable [17]. In practice, an instrument is said to be weak if the F-statistics for testing the nullity of parameter *β*_2_ in the second model is inferior to 10 [18].

Another equivalent way to calculate the IV estimates (but without their standard errors!) is to perform a “two-stage least squares”, regressing *X* on *Z* in a first stage (this is the second model above), and regressing *Y* on the obtained fitted values *X̂*(*Z*) in a second stage. The method of instrumental variables can be readily extended to the case of several instrumental variables (and therefore to the case of a qualitative instrument), which may be useful to improve the precision of the instrumental variable estimate. One can also adjust for additional covariates in each of the above models.

In addition to test for the nullity of the causal effect *β*_1_, one may also test for the absence of correlation between *X* and the error term in the first model, implying the equality of the parameters *β*_1_ and $\beta_{1}^{LS}$, using the Durbin-Wu-Hausman test. This may be of some interest when comparing several candidate models which may have generated the data (see the simulations below).

## 4. Simulations and Example

To illustrate that the method of instrumental variable is effective, we simulated data from five models consistent with the five above-mentioned interpretations (Table 1). In each case, we simulated an instrument *Z* satisfying the conditions. For simplicity, we took all intercepts in these models to be 0, all slopes to be 1, and the variables which were generated at each step were taken to be N(0,1), *i.e.*, normally distributed with mean 0 and variance 1.

**Figure 2.**
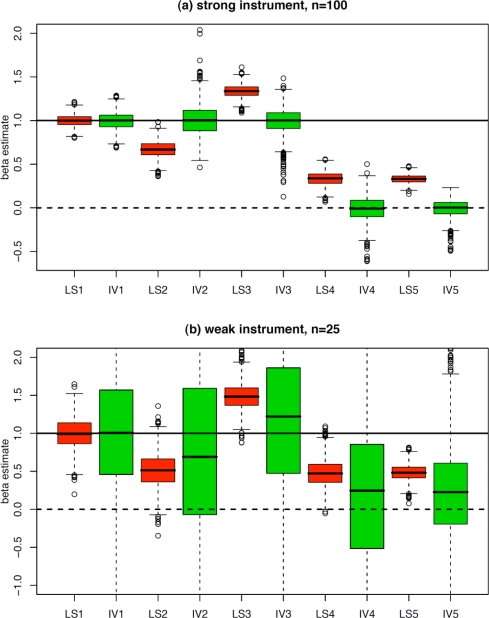
Results of simulations based on 1,000 samples under each of the five models described in Table 1, using (a) a strong instrument and n = 100 and (b) a weak instrument and n = 25. Shown are boxplots of the 1,000 least squares estimates (LS1, LS2, LS3, LS4, LS5, red boxes) and of the 1,000 instrumental variable estimates (IV1, IV2, IV3, IV4, IV5, green boxes) of the causal effect. The true causal effect to be estimated was *β*_1_ = 1 (solid horizontal line) for the first three models, and *β*_1_ = 0 (dashed horizontal line) for the last two models.

The causal effect of *X* on *Y* that we are looking for is *β*_1_ = 1 under the first three models, and is *β*_1_ = 0 under the last two models. Boxplots of the least squares (LS) estimates and of the instrumental variable (IV) estimates of parameter *β*_1_ obtained from 1,000 samples of size n = 100 under each of the five models are shown on the top panel of Figure 2. The LS estimate is unbiased under the first model, is consistently too small under the second model, and is consistently too large under the last three models. By contrast, the IV estimate is almost unbiased under each of the five models, which is actually remarkable. One can also notice that the IV estimate shows a higher variability than the LS estimate, which is the price to pay for correcting the bias of the latter. The Durbin-Wu-Hausman test was significant in 4.1% (which was close to the nominal 5% level) of the samples generated from the first model, for which $\beta_{1}=\beta_{1}^{LS}$ holds, in 66% of the samples generated from the second model, for which $\beta_{1}>\beta_{1}^{LS}$ holds, and in 88%, 90% and 100% from the samples generated respectively from the third, fourth and fifth models, for which $\beta_{1}<\beta_{1}^{LS}$ holds.

To provide an idea of what may happen when using a weak instrument, we considered the same five models, but the slopes involving *Z* were set to 0.25 (instead of 1) in each model. In addition, we reduced the sample size to n = 25. Under that setting, the F-statistic in the first stage regression was smaller than 10 in more than 95% of the generated samples. Boxplots of the estimates obtained from 1,000 samples are shown on the bottom panel of Figure 2. One can see that the variance of the IV estimates dramatically increased (compared to the top panel), while some non-negligible bias appeared.

We next provide an example with real data to illustrate that the method of instrumental variable is able to correct for the bias of least squares in a case of reverse causation. We used the 1,268 participants of the population-based CoLaus study [19], who reported that they consumed alcohol regularly and who had available data for genetic markers located with the gammaglutamyl transferase 1 (*GGT1*) gene as well as circulating GGT levels (*X*). CoLaus participants have been genotyped using the Affymetrix 500 K chip, alcohol consumption was assessed using a standardized questionnaire and coded in units of alcohol per week, and GGT levels were measured using standard procedures as previously described [19]. As we were interested in exploring an example of reverse causation, we chose *Y* to be the reported alcohol consumption and tested whether circulating GGT (*X*) could cause alcohol consumption (which we know is the opposite of the reality) using the best *GGT1* marker as our instrument (*Z*). Rs2017869 explained 1.12% of circulating GGT levels. The parameter *β*_1_ was estimated using least squares and the method of instrumental variables (the latter with the ivregress 2sls command implemented in Stata 10.0). The LS estimate (95%CI) was 5.53 (4.73;6.33) mmol/L per risk allele. The IV estimate (95%CI) was −4.60 (−13.82; 4.63) mmol/L per risk allele, which was significantly different from the LS estimate in a Durbin-Wu-Hausman test (P = 0.03), and not significantly different from zero. Thus, while the result provided by least squares was highly significant, the instrumental variable approach did not show any evidence for a positive causal association of GGT on alcohol consumption.

## 5. Review of Observational Studies Using Mendelian Randomization

We searched MEDLINE using the following «Mendelian randomization» OR “Mendelian randomisation”, which retrieved 99 citations (January 13, 2009). We acknowledge that this search strategy might not have retrieved all publications using the concept of Mendelian randomization, but it should provide a good overview of what has been published. The aim was to identify original articles reporting results from an observational study using a Mendelian randomization approach. We also searched references from review papers and original articles, as well as citations of these papers.

**Figure 3.**
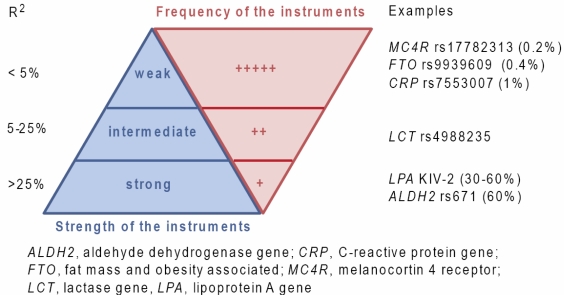
Type and frequency of genetic instruments in Mendelian randomization. R^2^ represent the proportion of variance of *X* explained by *Z*. Percentage in parentheses represent R^2^ value for the first linear regression in 2-stage least squares regression models.

We identified 23 studies with a dichotomous trait as the outcome of interest (Table 2) and 15 studies with a continuous trait as the outcome of interest (Table 3). Considering that the instrumental variable approach has been introduced, and is well understood, for a continuous outcome, it was a bit of a surprise to find that a majority of studies in fact applied this method to a dichotomous outcome (using non-linear models and odds-ratios to quantify the associations, for which the method has not been quite validated, see also the next section). Thirteen out of 23 studies focusing on binary outcomes (Table 2) reported results compatible with a causal association. Most studies were in the field of cardiovascular epidemiology and cancer epidemiology. For continuous outcomes (Table 3), half of studies reported some evidence for causality and most studies were in the field of cardiovascular epidemiology. Most instruments reported in these studies were weak (Figure 3). We also found many studies that claimed to use a Mendelian randomization approach although they only analyzed the genotype-outcome association, hence focusing on hypothesis testing (*i.e*., to confirm or disprove causality). Yet, what is of interest in the Mendelian randomization approach is to estimate the causal effect of *X*, the modifiable factor, on *Y* and not simply the association between *Z* and *Y*.

## 6. Some Limitations of Mendelian Randomization

In order to use Mendelian randomization to infer causality in observational epidemiology, numerous conditions need to be fulfilled [13,55–57]. A major limitation of this approach is that it is difficult, in practice, to me*et al.*l these conditions for a given risk factor—outcome association. To fulfill the first condition, *Z* and *X* should be correlated (genetic instruments for common complex diseases are typically quite weak). This indirectly implies that there is some level of allelic homogeneity (*i.e*., common variants rather than rare variants). Note that for many exposures, no suitable genetic instrument is available. The second and third conditions are the problematic ones. They state that *Z* is (marginally) independent from all potential confounders *U*, and that *Z* and *Y* are independent conditionally on *X* and *U* [57]. In an excellent introduction to Mendelian randomization, Didelez and Sheehan [58] wrote that “if we know a gene closely linked to the phenotype without direct effect on the disease, it can often be reasonably assumed that the gene is not itself associated with any confounding factors”. See however Section 7 of that paper for situations in which these conditions are not satisfied. Mendel’s second law (*i.e*., the law of independent assortment of alleles at the time of gamete formation) is not always true in that genetic variants located on the same chromosome, particularly for close loci, do not segregate independently (*i.e*., they are linked), as detailed in Lawlor *et al.* [13]. At the population level, such physical linkage patterns result in linkage disequilibrium, *i.e*., correlations between alleles at nearly loci. In genetic epidemiology, the second condition implies, among others, that there should be no confounding due to linkage disequilibrium (*i.e*., instrument *Z* should not be correlated with other genetic variants having an effect on the outcome of interest, *Y*) [13]. However, the instrument *Z* does not necessarily need to be causally associated with X, in that another genetic variant associated to both *Z* and *X* might be the true causal variant [13]. Similarly, population stratification, *i.e.,* the existence of population subgroups with different allele frequencies and outcome distributions, may violate this second condition as well. In the Mendelian randomization context, confounding may exist if the subgroups (these often correspond to ethnic groups) are associated to both *Z* and *Y* [13].

Also, there should be no pleiotropy, (*i.e*., *Z* having multiple effects, which do not pass through *X*). This is however only a problem if the other functions of *Z* are associated to *Y* [13]. There should be no canalization (also called developmental compensation), which corresponds to a functional adaptation to a specific genotype influencing the expected genotype-disease association [13]. For instance, a gene expressed during fetal development may enhance the expression of other genes having compensatory effects on the outcome [13]. For most genetic variants involved in complex traits, the effect size is small and we do not know if such modifications would lead to developmental compensation. Furthermore, there should be no segregation distortion at the locus of interest. Although unlikely, it has been reported that some loci in the human genome show some evidence of such distortion [59]. Of course, there should be no selective survival due to the genetic variant of interest. Considering that the randomization occurred many years before the analysis is conducted, if a specific genotype were associated with increased early mortality, the genotypic distribution at the time of the study might not reflect the initial distribution. For instance, the *C677T MTHFR* variant has been associated with fetal viability [60,61]. And finally, although this has rarely been assessed so far, there should be no parent-of-origin effect (*i.e*., the effect of the paternally transmitted allele should be the same as the effect of the maternally transmitted allele).

A practical condition is that there should be enough data to establish reliable genotype-intermediate phenotype, or genotype-outcome, associations. In our literature review, we observed that for many publications, estimates for these two associations came from different studies. Whenever independent studies have analyzed these two relationships, separate meta-analyses can be conducted. For studies having assessed both relationships, a multivariate model is needed in order to take into account the correlation in the genotype–phenotype and genotype–disease associations. Minelli *et al.* proposed a method to use meta-analysis results in a multivariate Mendelian randomization approach [62,63]. Note that their approach is based on odds ratios (see below). According to some authors, the advantage of using the same study (or studies) to estimate both associations include (1) being in a better position to examine whether or not the assumptions underlying the instrumental variable method have been violated or not and (2) having greater precision [13].

Many of the studies we identified applied a Mendelian randomization approach with a binary outcome. While econometricians have proposed instrumental variables methods for binary outcomes (see Lawlor *et al.* [13] for a nice review), the generalization of instrumental variables to non-linear systems is not at all straightforward and may require additional assumptions [13,58]. One possibility is to build a linear model using risk differences, instead of risk ratios [64]. Another is to use a latent model, in which the underlying outcome variable is assumed to be continuous and the observed binary outcome reflects whether or not a specific threshold has been reached (e.g., probit models). Log-linear and logistic structural mean models for binary outcomes were also developed [65,66], where it was not possible to avoid some bias. Palmer *et al.* [67] proposed an adjusted IV estimate to reduce the bias of the classical IV estimate applied to a binary outcome, but admitted to ignore whether, and under what conditions, the estimated parameter had a strictly causal interpretation. They also noted that “instrumental variable theory has not been fully generalized to non-linear situations”. Finally, one may obtain bounds on the causal effect using a non-parametric method whenever the instrument, the risk factor and the disease are all categorical [58]. Note that none of the published studies of binary outcomes we found used these methods.

## 7. Conclusions

The Mendelian randomization approach in observational epidemiology is a valuable tool that has taken a new dimension in the post-genomic era and is being used increasingly. This approach conceptually relies on an instrumental variable approach. There have been some successes of the Mendelian randomization approach to help unraveling causal relationships in observational epidemiology. Examples are the recently published evidence for the causal role of body mass index on blood pressure [45] or accumulating evidence against the causal role of CRP in coronary heart disease [26–28] or atherosclerosis [49,50]. This method however suffers from several limiting factors. First, most genetic variants (*Z*) only explain a very small proportion of variance of the phenotype of interest (*X*). This implies that very large sample sizes are usually needed (>10,000) to reach sufficient power. Second, for many associations of interest, it is not possible to find an appropriate instrumental variable. However, as many more instruments are being discovered, the prospects are improving. Third, the success of this method heavily rests on the existence of allelic homogeneity, *i.e*., a common causal allele is shared by many individuals. Fourth, whereas analytic methods have been described for continuous outcomes, it is unclear to what extent these methods also apply to dichotomous outcomes. Considering the clear interest for epidemiologists to apply this concept for dichotomous outcomes such as diseases, it would be important, and even urgent, to clarify the issues on the validity of the instrumental variable approach in this context. More methodological development is needed before the instrumental variable approach can be confidently used for binary outcomes.

## Figures and Tables

**Table 1.** Description of the five models used for the simulations in Section 4.

**Model**	**Step 1**	**Step 2**	**Step 3**	**Step 4**	**Step 5**
1. Causal effect of X on Y	Z = N(0,1)	X = Z+N(0,1)	Y = X+N(0,1)		
2. Causal effect of X on Y and measurement errors on X and Y	Z = N(0,1)	Xtrue = Z+N(0,1)	Ytrue = Xtrue+N(0,1)	X = Xtrue+N(0,1)	Y = Ytrue+N(0,1)
3. Causal effect of X on Y and presence of a confounder	Z = N(0,1)	U = N(0,1)	X = Z+U+N(0,1)	Y = X+U+N(0,1)	
4. No causal effect between X and Y and presence of a confounder	Z = N(0,1)	U = N(0,1)	X = Z+U+N(0,1)	Y = U+N(0,1)	
5. Causal effect of Y on X (reverse causation)	Z = N(0,1)	Y = N(0,1)	X = Z+Y+N(0,1)		

**Table 2.** Literature review for dichotomous outcomes analyzed using a Mendelian randomization approach.

**Outcome (Y)**	**Gene**	**Variant(s) (Z)**	**Risk factor (X)**	Causality*	**Reference**
**Cardiovascular epidemiology**					
Type 2 diabetes	*SHBG*	rs1799941	SHBG	+	[20]
Type 2 diabetes	*SHBG*	rs6257, rs6259	SHBG	+	[21]
Type 2 diabetes	*BCMO1*	rs6564851	β-carotene	−	[22]
Type 2 diabetes	*MIF*	rs1007888	MIF	+	[23]
- Coronary artery disease	*LDLR*	rs2228671	LDL-cholesterol		[24]
Coronary heart disease	*PCSK9*	Y142X, C679X	LDL	+	[25]
Coronary heart disease	*CRP*	rs1130864	CRP	−	[26]
Coronary heart disease	*CRP*	rs7553007	CRP	−	[27]
Myocardial infarction	*CRP*	rs1130864	CRP	−	[28]
Myocardial infarction	*LPA*	KIV-2 (CNV)	Lp(a)	+	[29]
Myocardial infarction	*FGB*	−148C/T	fibrinogen	−	[30]
-Stroke	*MTHFR*	C677T	homocysteine	+	[31]
Hypertension	*CRP*	rs1800947,	CRP	−	[32]
Metabolic syndrome	*LCT*	rs4988235 (-13910-C/T)	Milk consumption	+	[33]
Hypertriglyceridemia	*RBP4*	rs3758538	RBP4	−	[34]
**Cancer epidemiology**					
Cancer	*APOE*	E2, E3, E4	cholesterol	−	[35]
Head and neck cancer	*ALDH2*	rs671	Alcohol consumption	+	[10]
Oesophageal cancer	*ALDH2*	rs671	Alcohol consumption	+	[9]
Lung or kidney cancer	*FTO*	rs9939609	BMI	+	[36]
**Other topics**					
Polycystic ovary syndrome	*IRS-1*	Gly972Arg	Insulin	+	[37]
Depression	*PON1*	rs662	PON1 activity	−	[38]
Stillbirth	*CYP1A2, NAT2, GSTA1*	slow/fast metabolizers	caffeine	+	[39]
Cataract	*FTO*	rs9939609	BMI	+	[40]

* + means evidence for causality; − means no evidence for causality. SHBG, sex hormone binding protein; LDL, low density lipoprotein; CRP, C-reactive protein; Lp(a), lipoprotein a; RBP4, retinol binding protein 4; BMI, body mass index; PON1, paraoxonase 1 Gene symbols: *SHBG,* sex hormone-binding globulin; *PCSK9*, proprotein convertase subtilisin/kexin type 9. *CRP*, C-reactive protein; *LPA*, lipoprotein, Lp(a); *FGB*, fibrinogen beta chain; *LCT*, lactase; *RBP4*, retinol binding protein 4; *APOE,* apolipoprotein E*; ALDH2,* aldehyde dehydrogenase 2 family (mitochondrial); *FTO,* fat mass and obesity associated; *IRS-1,* insulin receptor substrate 1; *PON1,* paraoxonase 1; *CYP1A2,* cytochrome P450, family 1, subfamily A, polypeptide 2; *NAT2*, N-acetyltransferase 2 (arylamine N-acetyltransferase); *GSTA1*, glutathione S-transferase alpha 1. *MIF,* macrophage inhibitory factor; *MTHFR*, methylene tetrahydrofolate reductase; *BCMO1*, beta-carotene 15,15’-monooxygenase 1.

**Table 3.** Literature review for continuous outcomes analyzed using a Mendelian randomization approach.

**Outcome (Y)**	**Gene (s)**	**Variant(s) (Z)**	**Risk factor (X)**	Causality*	**Reference**
Metabolic traits (insulin, lipids, etc)	*FTO*	rs9939609	BMI	+	[41]
BMI	*CRP*	rs1800947, rs1205	CRP	−	[42]
BMI	*CRP,*	rs7553007	CRP	+	[43]
	*LEPR*	rs1805096			
BMI, blood pressure, triglycerides, HDL, waist-to-hip ratio, HOMA-R	*CRP*	rs1800947, rs1130864, rs1205	CRP	−	[44]
Blood pressure	*MC4R*	rs17782313 rs9939609	BMI	+	[45]
	*FTO*				
Blood pressure	*CRP*	rs1800947,	CRP	−	[32]
Bone mass	*MC4R*	rs17782313	adiposity	+	[46]
	*FTO*	rs9939609			
Bone mass density, bone fractures	*LCT*	rs4988235 (-13910-C/T)	Calcium intake	+	[47]
HbA1c	*CRP*	rs1130864, rs1205, rs3093077	CRP	−	[48]
Carotid-intima media thickness	*CRP*	rs1130864, rs1205, rs3093077	CRP	−	[49]
Carotid-intima media thickness	*CRP*	rs 2794521, rs3091244, rs1800947, rs1130864, rs1205	CRP	−	[50]
Carotid-intima media thickness	*FTO*	rs9939609	BMI	+	[51]
Serum leptin	*CRP*	rs 2794521, rs3091244, rs1800947, rs1130864, rs1205	CRP	−	[52]
Lung function	*CRP*	rs1205, rs1800947	CRP	+	[53]
Physical functioning	*IL-18*	rs5744256	IL-18	+	[54]

BMI, body mass index. CRP, C-reactive protein. IL-18, interleukin 18. *LEPR*, leptin receptor. For other gene symbols, see Table 2 legend.
